# An exotic plant successfully invaded as a passenger driven by light availability

**DOI:** 10.3389/fpls.2022.1047670

**Published:** 2022-12-07

**Authors:** Yanyan Liu, Wenjun Li, Xiaolin Sui, Airong Li, Kaihui Li, Yanming Gong

**Affiliations:** ^1^ Bayinbuluk Grassland Ecosystem Research Station, Xinjiang Institute of Ecology and Geography, Chinese Academy of Sciences, Urumqi, China; ^2^ Chinese Academy of Sciences (CAS) Research Center for Ecology and Environment of Central Asia, Urumqi, China; ^3^ State Key Laboratory of Desert and Oasis Ecology, Xinjiang Institute of Ecology and Geography, Chinese Academy of Sciences, Urumqi, China; ^4^ Yunnan Key Laboratory for Wild Plant Resources, Department of Economic Plants and Biotechnology, Chinese Academy of Sciences, Kunming, China

**Keywords:** resource competition, invasive annual plant, species richness, species removal, fertilization, driver-passenger models

## Abstract

Invasive exotic plant species (IEPs) are widely distributed across the globe, but whether IEPs are drivers or passengers of habitat change in the invaded spaces remains unclear. Here, we carried out a vegetation and soil survey in 2018 and two independent field experiments (*Pedicularis kansuensis* removal in 2014 and 2015, and fertilization experiment since 2012) and found that the invasive annual *P. kansuensis* was at a disadvantage in light competition compared with perennial native grasses, but the successful invasion of *P. kansuensis* was due to the sufficient light resources provided by the reduced coverage of the native species. Conversely, nitrogen enrichment can effectively inhibit *P. kansuensis* invasion by increasing the photocompetitive advantage of the native species. s*P. kansuensis* invasion did not reduce species richness, but did increase plant community coverage, productivity and soil nutrients. Furthermore, the removal of *P. kansuensis* had little effect on the plant community structure and soil properties. Our results suggest that the passenger model perfectly explains the benign invasive mechanism of *P. kansuensis*. The invasion “ticket” of *P. kansuensis* is a spare ecological niche for light resources released by overgrazing.

## 1 Introduction

Due to the increasing globalization of agricultural economy, invasive exotic plant species (IEPs) have been widely spread by humans unconsciously, most of them becoming dominant species, which has had a profound impact on the invaded ecosystems (Vitousek et al., 1996; Sala et al., 2000; Didham et al., 2005). Examples of the consequences of IEPs are a decrease in ecosystem biodiversity with the loss of some native species (Wilcove et al., 1998), and marked alterations in soil nutrients and community productivity (Liu and van Kleunen, 2017; Le Roux et al., 2018). Therefore, some IEPs have captured the attention of ecologists because of their formation of expanded populations in low diversity stands (Levine et al., 2003; Vilà et al., 2011). IEPs may have a competitive advantage over native resident species for the exploitation of nutrients (Huenneke et al., 1990; Davis et al., 2000) or light (Xu et al., 2022) that natives are not able to tap (Sun et al., 2014). IEPs with different life forms from the native flora may alter the community structure and habitat heterogeneity of the invaded area, thereby changing the resources available to resident species (Jager et al., 2009; Molinari and D’Antonio, 2014). Despite recent advances in our theoretical understanding of how IEPs affect plant communities (Wardle and Peltzer, 2017; Catford et al., 2018; Zhang and van Kleunen, 2019), the long-term consequences of competition between native and exotic species often remain unclear (Mordecai et al., 2015), and the relative importance of competition and habitat change on the impact of the invasion to the native ecosystem is lacking in field experiments (Carboni et al., 2021).

Exotic plant invasions threaten biodiversity and ecosystem function with long-term ecological consequences (Buckley and Catford, 2016; Livingstone et al., 2020; Mahood et al., 2021). An increasing body of evidence confirms that IEPs markedly reduce the species richness of invaded plant communities around the world (Vilà et al., 2011; Bernard-Verdier and Hulme, 2015; Carboni et al., 2021). However, the invasion of exotic plants can also enhance species richness of the invaded plant communities where the ‘weak invaders’ establish at low density (Sax et al., 2005; Maron et al., 2014; Török et al., 2021). Similarly, IEPs modify the physical and chemical environment of the soil, which impacts on the composition and structure of the vegetation community in ways different from the original native plants (Schimel et al., 1991; Sardans et al., 2017). For instance, some studies indicate that substantially higher above-ground net primary productivity (ANPP) and soil nitrogen (SN) concentrations can be achieved after the establishment of IEPs in the native plant communities (Rout and Callaway, 2009; McLeod et al., 2016; Mahood et al., 2021). When invasive perennial plants dominate, they not only compete directly with the residents for resources, but also often have profound effects on local ecosystem processes (Vilà et al., 2011; Carboni et al., 2021). Conversely, invasive annual plants may be benign in the invaded plant community, especially when habitat disturbance makes them passively invasive rather than competitive (Bennett et al., 2016; Fahey et al., 2018).

Understanding the drivers of successful establishment and spread of IEPs is key to predicting new invasions in the context of global environmental change (Meyerson and Mooney, 2007; Gallien et al., 2019). Disturbed ecosystems are more likely to be successfully invaded by the IEP. Recent studies have shown that changes in soil nutrient availability or reduction of native species richness in a disturbed ecosystem can increase the susceptibility of the plant community to invasion (Schrama and Bardgett, 2016). However, community ecology theories generally emphasize competition as the primary driver of the impact of invasions on vegetation, whereas a combination of direct and indirect mechanisms is more likely to effectively explain the success and impact of invasions (Carboni et al., 2021). However, our knowledge of the mechanisms involved remains limited.

Nutrient enrichment can shift competition from nutrition to light (Grime, 1973). Light is a directional supply resource whose availability declines exponentially from the top of the vegetation canopy to the soil surface (Craine and Dybzinski, 2013), so taller, fast-growing individuals reduce the availability of light for slower-growing, shorter species or individuals (DeMalach et al., 2017). For example, in Hawaii’s montane rainforests, results showed that the invading exotic tree species, *Fraxinus uhdei* and *Morella faya*, increased forest biomass by 32.51%, which reduced light availability and resulted in very few understory species (Asner et al., 2008). Although changes of plant community in physical structure after invasions are rarely documented, they may have substantial effects on native plant species by altering key resources such as light availability (Molinari and D’Antonio, 2014). In different native communities, symbiotic plants of different sizes can avoid light competition by using light at different spatial or temporal scales, thus maximizing light resource utilization (Zuppinger-Dingley et al., 2014). However, it is unclear whether this mechanism applies to IEPs, especially in the case of nutrient enrichment and the competition for light between IEPs and native species of different life forms (Molinari and D’Antonio, 2014; Xu et al., 2022).

The diversity- invasibility hypothesis suggests that diverse communities should be more resistant to invasive species than less diverse ones (Elton, 1950). It is usually native symbiotic species that suppress the invasion of exotic species by using complementary resources to reduce the availability of limited resources (Tilman, 1999; Fargione et al., 2003; Fargione and Tilman, 2005). Thus, as native species richness increases, there will be fewer resources or niches for exotic species to exploit (Tarasi and Peet, 2017). However, the abundance of native species (such as in the coverage of plants in grassland ecosystems) can also result in a greater resistance to invasion from exotic species through light competition, but this is often ignored when discussing the mechanism of plant invasion (Bernard-Verdier and Hulme, 2015; Pearse et al., 2019).

It is important to recognize that exotic species may sometimes be passengers in an empty niche rather than drivers of habitat change (MacDougall and Turkington, 2005). Hence, there are different contributions of the IEPs and habitat disturbances to native species richness (or abundance) of plant communities. (Bernard-Verdier and Hulme, 2015). MacDougall and Turkington (2005) therefore described their driver-passenger model, the first direct test of whether invasive species are the drivers of community change, or merely passengers in an empty niche (Didham et al., 2005). In the driver model, there are strong biological interactions between the IEPs and native residents, and the dominance of the exotic species in the plant communities is a direct result of the competitive exclusion of the native species. The model predicts that experiments to remove IEPs will lead to the recovery of the native species. In the passenger model, habitat disturbance has a direct negative effect on native species, but the biological interaction between invasive and native species is weak or non-existent, so the IEPs have the advantage to fill the effectively vacant ecological niche (Didham et al., 2005; MacDougall and Turkington, 2005). Therefore, determining whether the invasion of exotic species conforms to the driving model or the passenger model can help identify the underlying cause of the invasion and predict its impact on the local plant community (MacDougall and Turkington, 2005).

Here, we investigated the invasion of *Pedicularis kansuensis* in the grasslands of the Bayinbuluk Alpine steppe, where it is considered to be the most problematic invasive species in the region (Liu et al., 2017; Li et al., 2019; Wang et al., 2019). Three independent field experiments (sample survey, *P. kansuensis* removal and fertilization experiment) were conducted to compare the invasive and non-invasive communities to verify the following hypotheses: (1) The invasion of *P. kansuensis* was facilitated by the spatial gap caused by habitat disturbance, rather than its competitive advantage over the native species. (2) Nutrient enrichment increased coverage of native species and indirectly excluded *P. kansuensis* invasion by the advantage of light competition. (3) The passenger model, rather than the driving model, could well reveal the dynamics of *P. kansuensis* invasion in the alpine steppe.

## 2 Materials and methods

### 2.1 Study area

Our studies were carried out near the Bayinbuluk Grassland Ecosystem Research Station, Chinese Academy of Sciences (42.88471581° N, 83.70756803° E). Located in the Bayinbuluk Basin of the Tianshan Mountains in northwest China, the Bayinbuluk Grassland is the second largest grassland in China, covering an area of approximately 23,000 km^2^ and is composed of typical alpine and dry grassland. The height above sea level is about 2500 m. Bayinbuluk Grassland is natural grassland for grazing in the spring and autumn. The mean annual precipitation is 300.8 mm, 77.6% occurring from May to August. The mean annual temperature is -4.8°C, being lowest in January (-26.7°C) and highest in July (11.6°C) (data provided by the Bayinbuluk, Tianshan Mountain meteorological station (Liu et al., 2017). The soil is classified as a Cambisol in the Food and Agriculture Organization (FAO) soil classification system, is high in organic matter and nitrogen but low in phosphorus. There are 36 plant species belonging to 26 genera and 16 families, with the perennials, *Festuca ovina* and *Stipa purpurea* as the dominant species.

*P. kansuensis* is an annual or biennial semi-parasitic forb with a flowering period from June to August, which is widely distributed in western China and Nepal and distributed separately in the Qinghai-Tibet Plateau and Tianshan Mountains (Li et al., 2019). It is reported that *P. kansuensis* has a high seed setting rate, is often distributed in clusters and can spread rapidly in a short period of time by seed reproduction, and is considered a strong invader (Sui et al., 2016; Liu et al., 2017).

### 2.2 Field experiment design, sampling and data collection

#### 2.2.1 The sample survey

Eight randomly chosen sites of 10 m × 10 m were surveyed in the Bayinbuluk steppe in early August 2018. The eight sites were GPS referenced with latitude, longitude, and elevation (eTrex Venture, Garmin, USA). The distribution of surveyed sites is presented in **Table S1**. At each site, native and invaded quadrats of 1 m^2^ were paired, with ≤ 5 m between them in order to minimize differences in soil characteristics caused by factors other than plant invasion. Three pairs of native and invaded quadrats were investigated at each site (a total of 48 quadrats in the eight sites). In each quadrat, the number of plant species was recorded and a ruler was used to measure the maximum plant height of each species. Coverage was evaluated within three fixed place quadrats at each site by the same person over time for consistency (Fahey et al., 2018).

After removing the litter layer, soil samples (0–20 cm) were randomly collected with three replicates at each quadrat using a hand auger. The three samples from each quadrat were mixed evenly after removing organic debris and stones. Forty-eight soil samples were collected using a hand auger at the eight sites. A small portion of each fresh soil sample were stored at 4°C immediately after collection to determine the initial gravimetric moisture content. This was determined later by comparing the soil weight before and after drying the samples at 75°C for 48 h to a constant weight.

After air-drying, the remaining soil samples were ground to a fine powder using a ball mill and passed through a 2-mm sieve (Retsch MM 400; Retsch, Haan, Germany). Soil organic carbon content (SOC) was measured by using the Mebius method with Walkley-Black acid digestion (Nelson and Sommers, 1982). Soil total nitrogen content (SN) was analyzed with a Kjeltec System 2300 Analyzer Unit (Tecator, Höganäs, Sweden). Soil total phosphorus content (SP) was determined with the molybdate/ascorbic acid blue method after digestion with HClO_4_ and H_2_SO_4_ acid (John, 1970). Soil total potassium content (SK) after digestion was determined by optical emission spectroscopy (ICP-OES 7000DV, Perkin Elmer, USA). Soil pH was determined from 50 ml of water extract of 10 g fresh soil using a pH meter (SevenExcellence-S470, USA) (Bao, 2000).

#### 2.2.2 Field experiment of *P. kansuensis* removal

The *P. kansuensis* removal experiment was established near the Bayinbuluk Grassland Ecosystem Research Station in early September, 2014 (for the location, see **Table S1**). Five 10 m ×10 m plots were selected from the 100 m ×100 m fenced area which was established at the start of the experiment for fixed-point continuous observation (2014-2015). In each 10 m ×10 m plot, 12 quadrats invaded by *P. kansuensis* were randomly selected (coverage of *P. kansuensis* was about 30%). Thereof, 6 quadrats with intact *P. kansuensis* and 6 quadrats of *P. kansuensis* removal were randomly assigned. The area of each sampling quadrat was 1 m × 1 m. In the quadrat of *P. kansuensis* removal, *P. kansuensis* were removed at the seedling stage in early June. *P. kansuensis* were removed every three days for a month to ensure that there were no *P. kansuensis* in the quadrat. Three quadrats were randomly selected from each of the two treatments for investigation in early September, 2014 and the remaining quadrats were investigated and analyzed in September, 2015.

The plant species were mowed at ground level in the selected quadrats, and the aboveground biomass of each species was air-dried and weighed after removing the litter and other impurities. Root biomass was obtained using a root drill (8cm diameter). The methods of the vegetation survey and soil sample acquisition and analyses are the same as described above for the sample survey.

#### 2.2.3 Field fertilization experiment

Another 100 m ×100 m fenced area was established for the fertilization experiment in 2011. Since 2012, nitrogen fertilizer (CO(NH_2_)_2_) and phosphate fertilizer (Ca(H_2_PO_4_)_2_) were added four times a year at the end of May, June, July and August until the end of the experiment. CO(NH_2_)_2_ and/or Ca(H_2_PO_4_)_2_ was mixed with 0.5 kg native soil that had been passed through a 1 mm sieve and heated at 120°C for 24 hr in an oven. The resultant fertilizers were spread at the site as evenly as possible by hand. This experiment was carried out according to Nutrient Network: A Global Research Cooperative, NutNet, (http://nutnet.science.oregonstate.edu).

Six treatments were set up in a randomized block experiment, namely CK (control treatment), N1 (193 g N 36m^-2^ yr^-1^), N2 (386 g N 36m^-2^ yr^-1^), N (771 g N 36m^-2^ yr^-1^), P (1464 g P 36m^-2^ yr^-1^) and NP (771 g N 36m^-2^ yr^-1^ and 1464 g P 36m^-2^ yr^-1^). Each treatment was replicated in 5 plots of 6 m × 6 m. The invasion of *P. kansuensis* was detected in the experimental area in 2018, and the scale of invasion reached a stable maximum in 2021, as indicated by the stable soil content of *P. kansuensis* seeds (1179 ± 243 seeds m^-2^ from our unpublished data). The soil environment, community structure and leaf traits at the community level were analyzed in the 10 years of fertilization and 4 years of *P. kansuensis* invasion to test our hypotheses.

The methods used for vegetation survey, soil sample acquisition and analyses are the same as described above for the sample survey.

For leaf sample analyses, at each quadrat, fresh mature foliar samples were taken from individual plants of each species and stored separately in paper bags. Foliar samples were obtained randomly from about 10 replicate plants of each species randomly. The foliar samples were rinsed with deionized water at least twice to reduce the influences of dust or soil, and the leaf area (LA) measured from photographs with IMAGEJ (Rasband 1997-2012, version 1.45a; source: http://imagej.nih.gov/ij). Then, the foliar samples were dried at 70°C to constant weight, and the dry mass (g) of 20-30 pooled leaves from replicate plants were determined with a precision of 0. 1 mg. The leaf dry matter content (LDMC) was obtained by dividing the leaf dry weight by leaf fresh weight. The specific leaf area (SLA) was calculated by dividing leaf area (cm^2^) by leaf dry mass (g).

Plant samples were dried at 105°C for 30 min using a portable drying oven in the field laboratory to minimize respiration and decomposition losses, and then transported to the Xinjiang Institute of Ecology and Geography, Chinese Academy of Sciences, and oven dried at 70°C to a constant weight. Dried plant materials were ground and passed through a 1-mm sieve (Retsch MM 400; Retsch, Haan, Germany). Leaf N concentration was analyzed with a PE-2400 CHN analyzer (Perkin-Elmer, Foster City, USA). Leaf P concentration was measured colorimetrically after H_2_SO_4_-H_2_O_2_-HF digestion using the molybdate/stannous chloride method (Kuo, 1996).

### 2.3 Statistical analysis

Data were tested for normality using the Kolmogorov-Smirnov test and for equality of error variance using Levene’s test. The data in the structural equation model were transformed logarithmically.

Collinearity was detected on LA, SLA, LDMC, LN and LP (VIF<10, refer to Quinn and Keough, 2002; See **Table S5**). To reduce the number of dimensions of community-level leaf traits, we performed a principal component analysis (PCA) on the five traits and used the first principal component (PC1, see **Figure S1**), which accounted for 98.27% of the total variation in the data, in the ensuing analyses of structural equation modeling involving community-level leaf traits. We then calculated the community-weighted mean trait values (FunTra) of PC1, weighted by the relative aboveground biomass of each dominant species in the community (Li et al., 2021).

**Figure 1:**
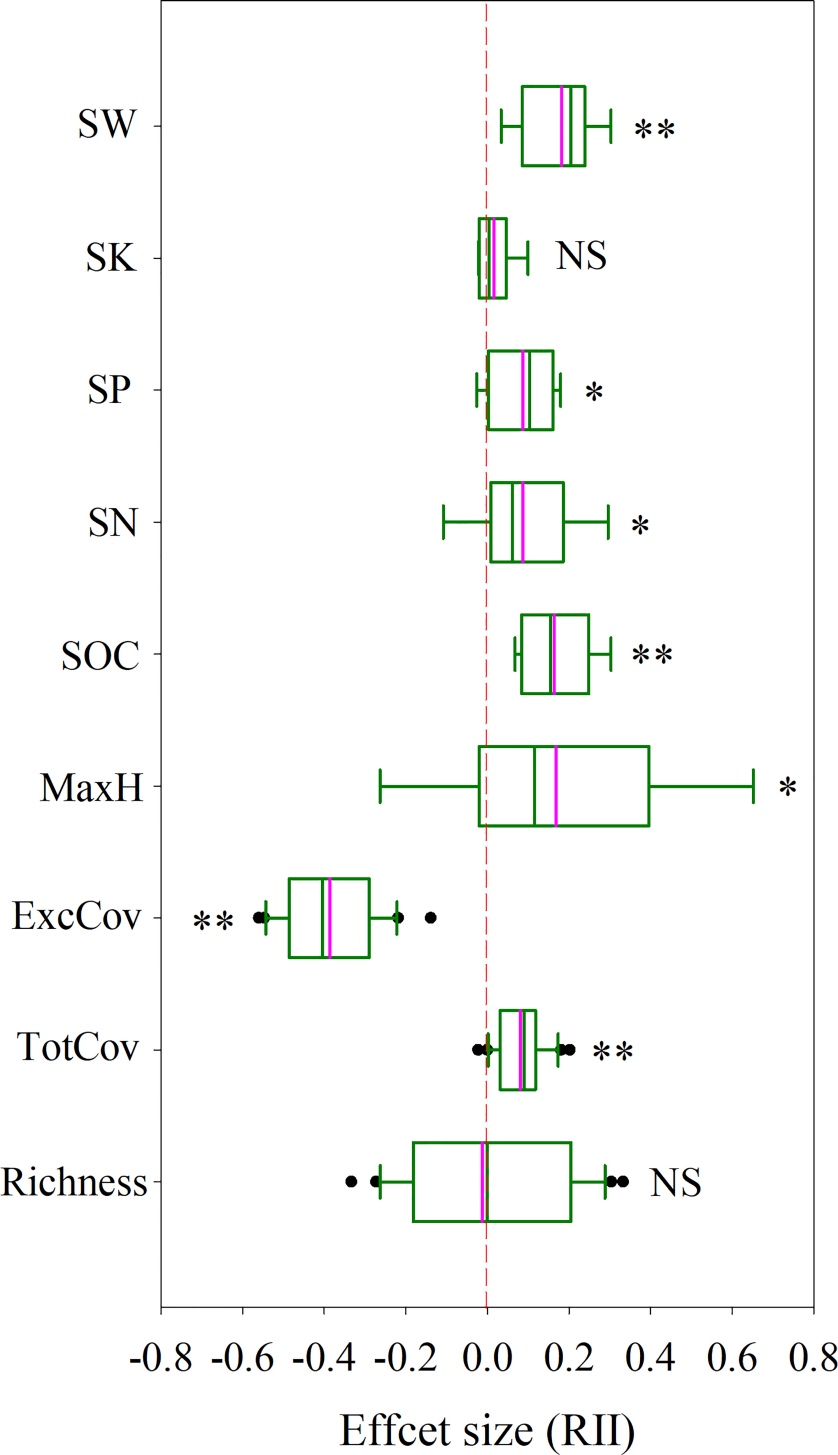
The effect size (RII) of *P. kansuensis* invasion on native community structure and soil environment in the sample sites. The pink line in the box diagram is the mean. Significant differences are reported from Mixed Linear Model as NS, P ≥ 0.05; *P < 0.05; **P < 0.01. Key: Richness, species richness; TotCov, total coverage of sample communities; ExcCov, coverage of sample communities excluding *P. kansuensis*; MaxH, maximum height of sample community; SOC, soil organic carbon content in sample sites; SN, soil total nitrogen content; SP, soil total phosphorus content; SK, soil total potassium content; SW, soil water content.

**Figure 2:**
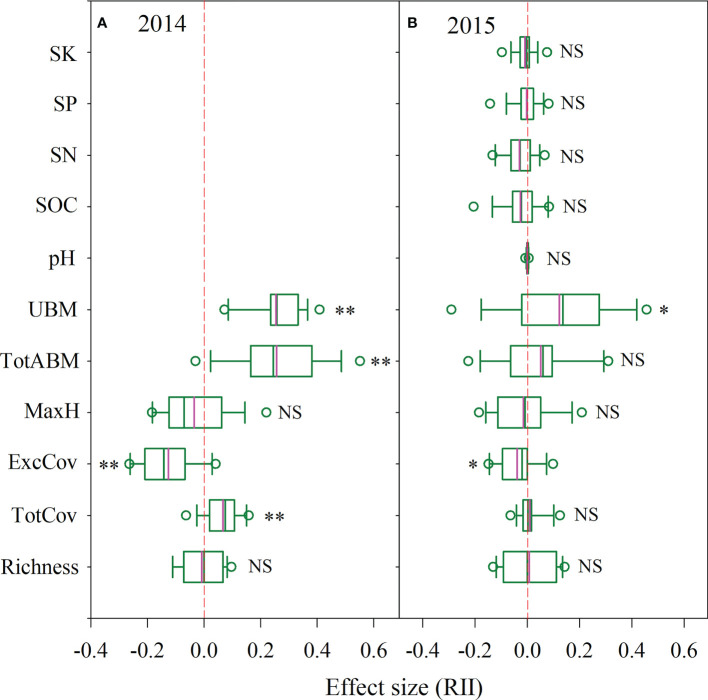
The effect size of *P. kansuensis* invasion on native community structure and soil environment by the field experiment of *P. kansuensis* removal [**(A)** 2014yr; **(B)** 2015yr]. The pink line in the box diagram is the mean. Significant differences are reported from Mixed Linear Model as NS, P ≥ 0.05; *P < 0.05; **P < 0.01. Key: TotABM, Total aboveground biomass of the sample plots; UBM, total underground biomass of the sample plots; pH, soil pH. For other abbreviations refer to the note in **Figure 1**.

A mixed linear model was used to compare the significance of the ecological parameters between invaded and native (or removal) sites in field experiments. In this model, the dependent variables were soil parameters and community indicators (**Figures 1**, **2**). The main fixed-effect explanatory variables were ecological parameters in the site (i.e. richness, SN), which was included both as a main effect and in interaction with the site-level variables, whereas the site was included as a random effect allowing slope (βC) and intercept parameters for invader parameter to vary for each site (Carboni et al., 2021).

To explore the effect of *P. kansuensis* invasion on the native community structure and soil environment, we calculated the relative interaction index (RII; Armas et al., 2004) for each paired native and invaded plot. RII was calculated as follows:


(1) $$RII=\frac{\left(Bw-Bo\right)}{Bw+Bo)}$$

where *Bw* is an ecological parameter (i.e. richness, coverage, maximum height, aboveground biomass, total underground biomass, soil organic carbon, soil total nitrogen, soil total phosphorus, soil total potassium, soil water content, and soil pH) of *P. kansuensis* invasion and *Bo* is the ecological parameter of the native community (or community of *P. kansuensis* removal). RII value ranges from -1 to 1. RII quantifies the relative change in the parameters of a native community as a result of *P. kansuensis* invasion. Thus, indicates how much and in which direction (i.e. positive, negative or neutral) the native community is affected by *P. kansuensis* invasion (Armas et al., 2004). Negative values indicate suppressive effects on the native plant community due to resource competition or other antagonistic effects from *P. kansuensis* invasion, whereas positive values indicate facilitative effects (i.e. increasing richness and aboveground biomass) by *P. kansuensis* invasion. Neutral values indicate that the invasion of *P. kansuensis* has little effect on the native community (Vink et al., 2017). In order to better quantify these impact categories, we divide RII values into three intervals as follows: RII values greater than 0.05 were regarded as positive; RII values less than -0.05 were regarded as negative; RII values between -0.05 and 0.05 were considered as neutral (Armas et al., 2004).

We further performed structural equation modelling (SEM) to disentangle the important pathways through which species richness and N addition influence the invader biomass.

Structural equation modeling (SEM) was used to disentangle and quantify the effects of SN, SP, species richness, coverage of native species and leaf traits at the community level (FunTra) on *P. kansuensis* invasion. The PCA was used to create a multivariate functional index (FunTra) to represent community weighted SLA, LA, LN, LP and LDMC. The first component (PC1) was then introduced as one new variable (FunTra) which weighted by the relative aboveground biomass of each dominant species in the community into the subsequent SEM analysis (Chen et al., 2016; Li et al., 2021). The goodness-of-fit of the final model was evaluated using the model χ^2^ test and the root mean square (RMS) error of approximation.

The above statistical analyses were conducted and presented using the statistical package SPSS (PASW statistics 25.0; IBM Corporation, Armonk, NY, USA) and the graphical package SigmaPlot 12.5 (SyStat Software Inc., San Jose, CA, USA). SEM analyses were conducted using AMOS 22.0 (Amos Development Corporation, Chicago, IL, USA).

## 3 Results

### 3.1 Effects of *P. kansuensis* invasion on the native community and soil environment

The results of the mixed linear model showed that total coverage (TotCov, **Table S2**) and the soil properties (excluding soil total potassium content, SK, which was not significant) in invaded sites were significantly higher than those in uninvaded sites (P<0.05) (**Figure 1**, **Table S3**). There were no significant differences in Richness and maximum height (MaxH) between invaded and non-invaded sites (P≥0.05). On the contrary, coverage excluded *P. kansuensis* (ExcCov) of invaded sites was significantly lower than total coverage in non-invaded sites (P>0.05).

Based on the sample survey, the relative interaction index (RII) for ExcCov was negative (**Figure 1**, RII = -0.39), whereas the RII values for Richness and SK were neutral and the RII values for the remaining parameters were positive, ranging from 0.071 to 0.18.

### 3.2 Effects of *P. kansuensis* removal on invaded communities and the soil environment

According to the mixed linear model analysis, in 2014, the TotCov, total aboveground biomass (TotABM) and underground biomass (UBM) in the invaded plots were significantly higher than that in the plots after *P. kansuensis* removal (removal plots hereafter) (TotCov, P=0.001, F=12.92, df=30; TotABM, P<0.001, F=23.74, df=30; UBM, P<0.001, F=59.24, df=30; **Figure 2A**, **Table S4**). The ExcCov of invaded plots was significantly lower than those of removal plots (ExcCov, P<0.001, F=24.22, df=30), whereas there was no significant differences in the Richness and MaxH (P≥0.05). In 2015, the UBM of invaded plots was significantly higher than those of the removal plots (P=0.026, F=5.47, df=30; **Figure 2B**, **Table S4**). The ExcCov of invaded plots was significantly lower than those of removal plots (ExcCov, P=0.015, F=6.71, df=30). There was no significant difference detected in the other parameters (P≥0.05).

After calculating RII values, in 2014, we found that the positive RII values included TotCov, TotABM and UBM (**Figure 2A**). Negative values included ExcCov and MaxH. Richness corresponded to neutral values. Conversely, in 2015, the RII values of more parameters were neutral (**Figure 2B**), with only TotABM and UBM as positive values.

### 3.3 The effects of ten-years fertilization on *P. kansuensis* invasion

**Figure 3:**
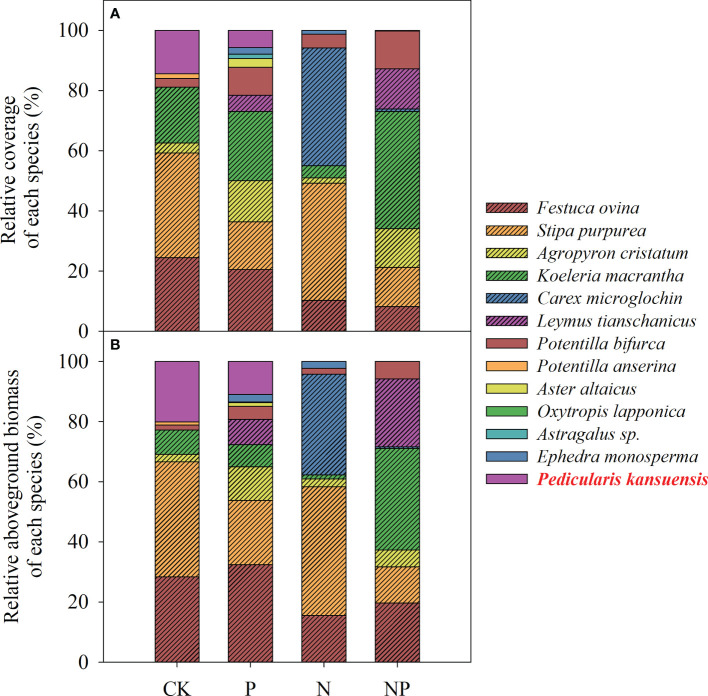
Relative coverage **(A)** and relative aboveground biomass **(B)** of each species in the sample plots given different fertilizers: CK, control treatments; P, phosphorus addition; N, nitrogen addition, including the N, N1, N2 treatments; NP, nitrogen and phosphorus addition.

Fourteen species were investigated in the sample plot of the fertilization experiment (**Figure 3**). Under different fertilization treatments, the relative coverage and relative aboveground biomass of *P. kansuensis* were different (**Figure 3**). Under the control and P fertilizer treatments, the relative coverage of *P. kansuensis* was 14.44% and 5.76% respectively, the relative aboveground biomass of *P. kansuensis* was 20.09% and 11.02%, respectively. However, no invasion of *P. kansuensis* was observed under N addition or N+P addition.

**Figure 4:**
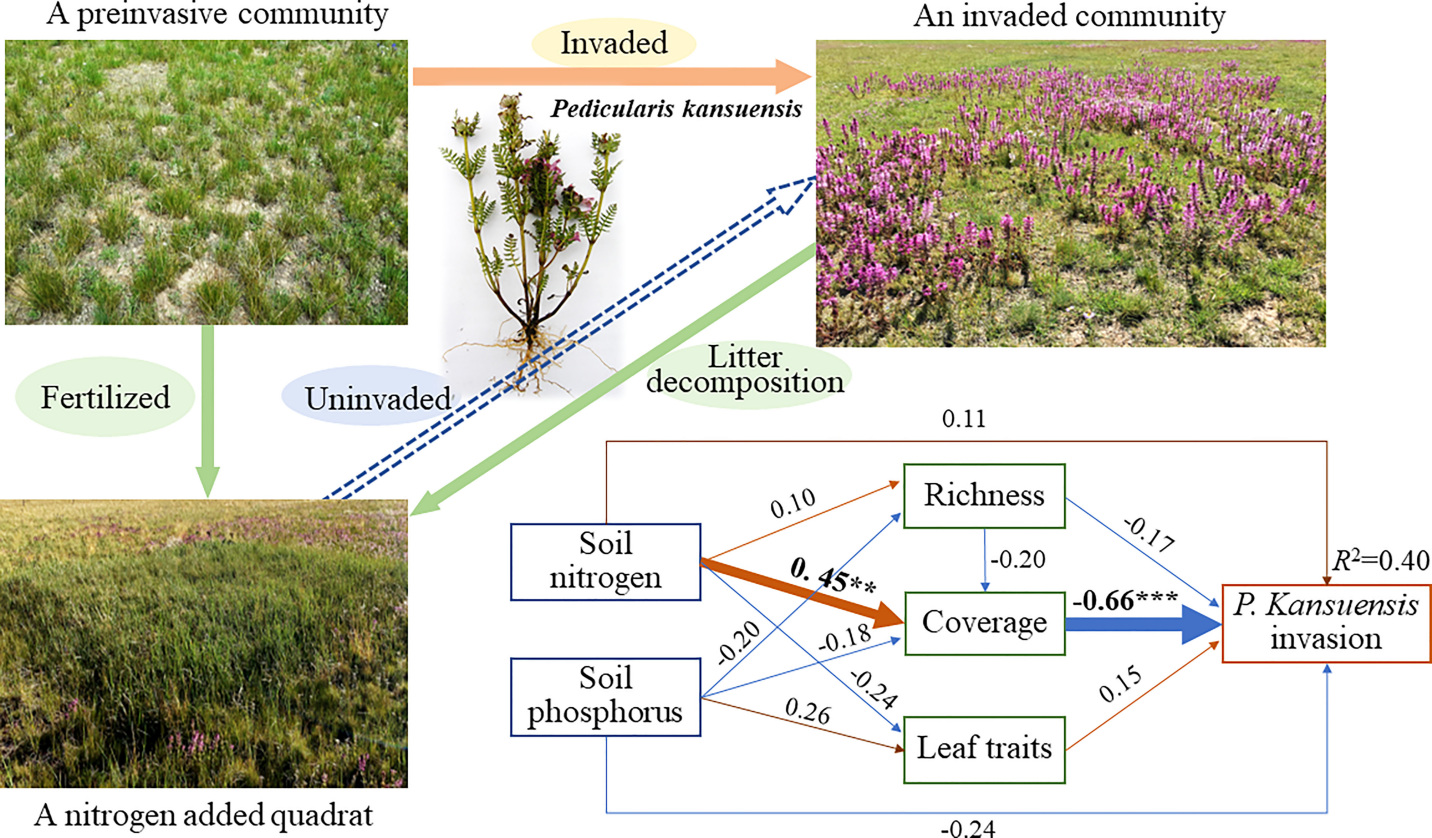
A schematic diagram showed the invasion process of *P. kansuensis*. Structural equation model for *P. kansuensis* invasion (aboveground biomass of *P. kansuensis*) of the fertilization experiment, is shown in the lower right panel and is based on the effects of soil nitrogen, soil phosphorus, species richness, coverage of sample communities excluding *P. kansuensis* (Coverage), community-weighted mean trait values (leaf traits). χ^2^ = 0.176, Df = 3, P = 0.981, CFI = 1.000, TLI = 2.003, RMSEA < 0.001, AIC = 48.176. Single-headed arrows indicate the hypothesized direction of causation. Orange and blue arrows indicate positive and negative relationships, respectively. The arrow width is proportional to the strength of the relationship. The numbers adjacent to arrows are standardized path coefficients, which reflect the effect size of the relationship. *R^2^* (coefficient of determination) indicates the proportion of variance explained by the relationship. Significance levels are as follows: **P < 0.01, ***P < 0.001.

The invasion of *P. kansuensis* was analyzed by SEM. The models explained 40% of the variances in *P. kansuensis* invasion (*P. kansuensis* aboveground biomass, P.K.ABM) with respect to the soil nitrogen (SN), soil phosphorus (SP), species richness, coverage of native species (ExcCov) and Leaf traits (FunTra) (**Figure 4**). Furthermore, SN exerted strong direct positive effects on ExcCov, with standardized path coefficients of 0.45 (**Figures 4** and **Figure S1**; P<0.01). ExcCov had significant negative effects on *P. kansuensis* invasion, with standardized path coefficients of -0.66 (**Figures 4** and **Figure S1**; P<0.001). Furthermore, SN and leaf traits had direct positive effects on *P. kansuensis* invasion, whereas SP and richness had direct negative effects on *P. kansuensis* invasion. There was a strong indirect negative effect (standardized path coefficients of 0.33) on *P. kansuensis* invasion by SN (**Figure S1**). In contrast, SP and richness had indirect positive effects on *P. kansuensis* invasion (Figure S1). In addition, the principal component analysis of five leaf traits showed that community weighted SLA was positively correlated with coverage and aboveground biomass of *P. kansuensis* (**Figure S2**).

## 4 Discussion

IEPs are often identified together with alterations to the soil environment of the area they invade (Ehrenfeld, 2003; Martin et al., 2017). However, it is difficult to determine whether the altered soil environment predates the invasion or is an effect of the invasion itself (Molinari and D’Antonio, 2014). Through the experiments of sample survey and *P. kansuensis* removal, our study confirmed that alterations to the soil environment was the result of *P. kansuensis* invasion, rather than the cause. *P. kansuensis* invasion significantly increased soil nutrients (i.e., SN, SP) and soil moisture. Our results are consistent with a growing body of evidence that differences in soil nutrients between invaded and non-invaded sites are primarily a result of plant invasion, rather than a facilitator for plant invasions (Hawkes et al., 2005; Allison et al., 2006; Castro-Diez et al., 2014; Kuebbing et al., 2014; Sardans et al., 2017; Zhou and Staver, 2019).

It has been shown that, invasive plants can cause significant changes in nutrient stocks and recycling rates through the deposition of their litter (Ehrenfeld, 2003; Liao et al., 2008; Carboni et al., 2021). Several studies have confirmed that rapidly increasing invasive species increase the input of nitrogen-rich litters (Allison et al., 2006; Liao et al., 2008; Sardans et al, 2017; Zhou and Staver, 2019). Increasing the overall input of high-quality litters can stimulate soil microbial activity and increase overall enzyme inputs (Allison and Vitousek, 2005; Kuzyakov, 2010). Increased enzyme activity may accelerate nutrient cycling, potentially creating a nutrient-rich soil environment (Zhou and Staver, 2019). For example, Zhou and Staver (2019) confirm that a nutrient-rich soil environment is beneficial to invaders and promotes their persistence, because invasive plants tend to be more resource-demanding and competitive than native species. However, the promoting influence of nutrient-rich soil environments on invasive plant species appears to favor invasive perennials, not invasive annuals (i.e. *P. kansuensis*). Nutrient enrichment promotes the growth of native grass plants and reduces the canopy density of the community, which in turn affects the germination, development, and reproductive failure of *P. kansuensis* (Liu et al., 2017). Our findings provide a possible mechanistic link between plant invasion and changes to ecosystem nutrient cycling (Zhou and Staver, 2019).

Increased resource availability and resource pulses tend to promote the invasion of IEPs (Besaw et al., 2011), but the relative importance of the increased resource supply to different life types of invaders may vary. In general, IEPs often adopt characteristic strategies associated with high resource requirements, and plant invasions are generally considered to depend on the availability or disturbance of site resources (Heberling and Fridley, 2016). For example, nutrients (especially nitrogen) and light availability are important factors in determining the outcome of the competition of *Phalaris* spp. with native US wetland species (Maurer and Zedler, 2002; Perry and Galatowitsch, 2004; Eppinga et al., 2011). When soil N availability is low, exotic plants are inhibited by native species, suggesting that native species are strong competitors for N (Perry and Galatowitsch, 2004). In contrast, Perry and Galatowitsch (2004) showed that light deprivation inhibited native species more than exotic species, suggesting that exotic species were strong competitors for light. However, our fertilization experiments confirm that the effects of nutrient (N) and light on *P. kansuensis* invasion were not isolated, but that soil nitrogen enrichment increased the coverage of native species and inhibited the invasion of *P. kansuensis* through photocompetitive advantage. Although *P. kansuensis* has characteristics associated with rapid growth and high biomass relative to native species (Liu et al., 2017), as a small-seeded invasive species, *P. kansuensis* usually requires light to germinate and may not survive in areas shaded by litter and dense perennial grasses (Molinari and D'Antonio, 2014; Sun et al., 2014). Thus, fertilization increased biomass and coverage of the native grass plants, thereby negatively affecting the recruitment of *P. kansuensis* seedlings into the plant community (Wang et al., 2018).

Like the results of most studies on plant invasions, *P. kansuensis* invasions inhibited the growth of native plants in that year by decreasing the coverage of native plants and aboveground biomass (such as grasses and legumes), but increased the height of native species in the invaded community (Kolb et al., 2002; Liao et al., 2008; Ehrenfeld, 2010; Jo et al., 2017; Ernst et al., 2021). The results showed that *P. kansuensis* was disadvantaged in light competition and that the success of their invasion was more due to grazing disturbance, which resulted in a reduced coverage of the native species. However, *P. kansuensis* invasion increased the total biomass (aboveground and underground) and coverage of the invaded plant community (Maron et al., 2014). Furthermore, the effect of *P. kansuensis* on alpine steppe vegetation was only reflected in the observed difference between the invaded and non-invaded communities from the sample survey, but not in the community changes after *P. kansuensis* removal, especially during the first-year after removal experiment. Our results showed that after the removal of invasive *P. kansuensis*, the resident plants could not utilize the exposed space and released resources, so there was no obvious improvement of the resident plant community. This further confirms our first hypothesis, that invasion of *P. kansuensis* was caused by the spatial gap caused by habitat disturbance, rather than its competitive advantage over the native species.

The introduction of IEPs is a major component of human-driven global change (Vilà et al., 2011; Pyšek et al., 2012; Carboni et al., 2021). One of the most common effects of plant invasions is a reduction in plant diversity and is generally quantified by species loss in invaded communities (Gaertner et al., 2009; Powell et al., 2011; Vilà et al., 2011; Pyšek et al., 2012). Nonetheless, our three independent field experiments confirmed that *P. kansuensis* invasion did not lead to a decrease in plant diversity (species richness), because as an annual plant, seed germination and growth of *P. kansuensis* in the coming year were affected by increased native plant cover due to the increase in soil nutrients by litter decomposition and prevented grazing by livestock.

In general, the higher the plant diversity, the denser the niche accumulation and the stronger the resistance to invasion, with species richness being the main indicator of diversity (Camill et al., 2004; Ernst et al., 2021). However, Ernst et al. (2021) suggest that excessive reliance on richness is a potential limitation in studies assessing the resistance to invasion, because the number of native species in a community may not be a good representation of the available niche space. The functional diversity of the community may have a more profound effect on the available niche space and on the invasibility of the community than taxonomic diversity (Wang et al., 2022). At the community scale, plant abundance (possibly coverage) is a better reflection of the available niche space and better explain the resistance to invasion (Ernst et al., 2021). Hejda et al. (2009) suggest that new invasions, which increase the dominance of the community by about 40-50%, have the most profound effects, especially if the invasive species are tall (e.g., *P. kansuensis*). In the Bayinbuluk Alpine steppe, the dominant exotic species are not annuals but perennials. Once invading perennial species are established, they continue to occupy the habitat (Kulmatiski, 2006; Norland et al., 2015; Kaul and Wilse, 2021) and resist the re-colonization from native species (Dickson et al., 2012; Martin et al., 2014). In contrast, annual invasive plants (such as *P. kansuensis*) are more benign to the invaded plant community.

The above conclusion was strongly verified in our fertilization experiment. In 2018, *P. kansuensis* began to invade our 10-year fertilization plots. Surprisingly, we observed that *P. kansuensis* invaded both the control and to a lesser degree, the phosphate fertilized plots. However, no invasions were observed in any of the four nitrogen-added plots (N, N1, N2 and NandP). This phenomenon further proved that the invasion of *P. kansuensis* was not driven by the rich soil nutrient resources. Instead, it is an invasion of unutilized niche space, which is confirmed by structural equation models. Furthermore, the decomposition of litter in invaded communities may inhibit the growth and development of *P. kansuensis* in the following year.

Although there is plenty of evidence that invasions may be the sole cause of significant ecological damage (e.g., Clavero and García-Berthou, 2005), but the question whether the exotic species is the driver of the ecological changes or merely a passenger should be asked in each new case of exotic invasion (Didham et al., 2005; King and Tschinkel, 2008; Ricciardi et al., 2013). In our study, the passenger model perfectly explained the invasion mechanism of *P. kansuensis* in the alpine steppe.

There is evidence that some native species have significantly improved coverage or productivity after the removal of alien dominant species (Didham et al., 2005). However, the removal of *P. kansuensis* had minimal impact on the local community structure and soil environment. We demonstrate that *P. kansuensis* invasions are passengers of the spatial resources of the community caused by long-term overgrazing, and that *P. kansuensis* did not displace native perennial grasses in the invaded community, but were weaker competitors (only reduced coverage of native species). A similar study by MacDougall and Turkington (2005) found that exotic dominant species are mainly passengers of long-term fire suppression, and thrive in conditions where perennial herbs are naturally abundant but easily replaced by repeated disturbances. Furthermore, the formation of persistent seed banks is thought to be more important for the persistence of annual species than perennials (Gioria et al., 2020; Gioria et al., 2021), and the invasion rate of small-seeded species is higher (Pyšek et al., 2009). As passengers of the alpine grassland, *P. kansuensis* ride time is the growing season (From May to September), and then remains in the invaded community in the form of a huge soil seed bank. Whether a large scale increase in the *P. kansuensis* population occurs in the following year depends on the coverage of the local dominant grass, because *P. kansuensis* is at a disadvantage in light competition. Once *P. kansuensis* enters the reproductive growth stage, it has a greater coverage advantage, but the premise is that there is sufficient space for seed germination and plant growth. Thus, as a passenger, the “ticket” of *P. kansuensis* invasion is a relatively barer soil surface in the plant community caused by overgrazing disturbance.

## 5 Conclusion

Our study provides new empirical evidence that the successful invasion of annual *P. kansuensis* can be attributed to the reduction of native species coverage caused by grassland degradation, the benefit of photocompetition, and that the invasion is benign. It is worth mentioning that this benign invasion means that *P. kansuensis* is not a transformer invasive species (Catford et al., 2012). Our results confirm the passenger model well, that invasion and removal of *P. kansuensis* have no significant impact on the local plant community and soil environment. We suggest that the large-scale invasion of *P. kansuensis* can be restrained by establishing a system to reduce over-grazing and the application of soil nitrogen fertilizer to promote the recovery of local grasses. In the future, an important direction is to extend our research results to the regional level with the use of remote sensing data, so as to evaluate the impact of spatial and temporal changes of environmental conditions on the distribution of *P. kansuensis* populations in the whole landscape and to further verify the passenger model.

## Data availability statement

The original contributions presented in the study are publicly available. This data can be found here: https://doi.org/10.6084/m9.figshare.20378049.v1.

## Author contributions

AL and YL conceived the ideas and designed the methodology; YG and YL collected and analysed the data and led the writing of the manuscript. All authors contributed critically to the drafts and gave final approval for publication.
