# Supplementary material for: An exotic plant successfully invaded as a passenger driven by light availability
Source: Front Plant Sci. 2022 Dec 7;13:1047670. doi: 10.3389/fpls.2022.1047670 (PMC9767969; doi:10.3389/fpls.2022.1047670)
Supplement: Supplementary file 1 [file DataSheet_1.docx]

**An exotic plant successfully invaded as a passenger driven by light availability**





FIGURE S1. Standardized direct (a) and indirect (b) effects derived from the corresponding structural equation model. SN, soil total nitrogen; SP, soil total phosphorus; Richness, species richness of sampling plots; Leaf traits, community-weighted mean trait values; Coverage, coverage of sample communities excluding *P. kansuensis*.

FIGURE S2. Principal component analysis of the relationships between leaf traits in the community scale, coverage and aboveground biomass of *P. kansuensis*.

Table S1. The geographical distribution of the sampling sites

| Sampling sites | | Longitude | Latitude | Height above sea level |
| --- | --- | --- | --- | --- |
| Field transect survey | 1 | 82.888319 | 42.868621 | 2570 |
|  | 2 | 83.680126 | 42.899457 | 2489 |
|  | 3 | 84.425209 | 42.835368 | 2417 |
|  | 4 | 84.538411 | 42.797188 | 2528 |
|  | 5 | 84.456462 | 42.687566 | 2403 |
|  | 6 | 83.990367 | 42.718905 | 2410 |
|  | 7 | 83.718601 | 42.876408 | 2464 |
|  | 8 | 84.086365 | 42.978575 | 2455 |
| Removal of *Pedicularis kansuensis* experiment | | 83.700699 | 42.891171 | 2500 |
| Fertilization experiment | | 83.705769 | 42.880376 | 2500 |

Table S2. The abbreviations of the variables and functional traits used in the analysis

| Number | Description | Abbreviation |
| --- | --- | --- |
| 1 | Species richness of a quadrat | Richness |
| 2 | Total coverage | TotCov |
| 3 | Coverage excluded *P. kansuensis* | ExcCov |
| 4 | Maximum height | MaxH |
| 10 | Total abovegound biomass | TotABM |
| 11 | Underground biomass | UBM |
| 12 | Specific leaf area | SLA |
| 17 | Leaf nitrogen content | LN |
| 18 | Leaf phosphorus content | LP |
| 21 | Leaf dry matter content | LDMC |
| 22 | Community-weighted mean trait value | FunTra |
| 26 | Soil pH (1:5) | pH |
| 27 | Soil organic carbon content | SOC |
| 28 | Soil total nitrogen content | SN |
| 29 | Soil total phosphorus content | SP |
| 30 | Soil total potassium content | SK |

Table S3. The results of mixed linear model for each index investigated in the sample survey. Refer to Table S2 for abbreviations in the table.

|  | df | F | P values |
| --- | --- | --- | --- |
| Richness | 44 | 0.16 | 0.694 |
| TotCov | 44 | 19.50 | < 0.001 |
| ExcCov | 44 | 186.71 | < 0.001 |
| MaxH | 44 | 4.57 | 0.038 |
| SOC | 8.00 | 22.92 | 0.001 |
| SN | 8.00 | 5.95 | 0.041 |
| SP | 8.00 | 8.53 | 0.019 |
| SK | 8.00 | 1.12 | 0.321 |
| SW | 8.00 | 24.95 | 0.001 |

Table S4. The results of mixed linear model for each index from the experiment of *P. kansuensis* removal. Refer to Table S2 for abbreviations in the table.

|  | 2014 yr | | |  | 2015 yr | | |
| --- | --- | --- | --- | --- | --- | --- | --- |
|  | df | F | P values |  | df | F | P values |
| Richness | 30 | 0.18 | 0.676 |  | 30 | 0.11 | 0.744 |
| TotCov | 30 | 12.92 | 0.001 |  | 25 | 0.82 | 0.372 |
| ExcCov | 30 | 24.22 | <0.001 |  | 25 | 6.71 | 0.015 |
| MaxH | 30 | 1.75 | 0.196 |  | 30 | 0.23 | 0.632 |
| TotABM | 30 | 23.74 | <0.001 |  | 25 | 1.01 | 0.323 |
| UBM | 30 | 59.24 | <0.001 |  | 25 | 5.47 | 0.026 |
| pH |  |  |  |  | 25 | 0.30 | 0.590 |
| SOC |  |  |  |  | 25 | 1.28 | 0.270 |
| SN |  |  |  |  | 25 | 1.95 | 0.175 |
| SP |  |  |  |  | 25 | 0.05 | 0.833 |
| SK |  |  |  |  | 25 | 0.65 | 0.427 |

Table S5 Collinearity test results of the fertilization experiment data on the PCA

|  | t | P | Tolerance | VIF |
| --- | --- | --- | --- | --- |
| LA | 1.62 | 0.12 | 0.80 | 1.24 |
| SLA | -1.81 | 0.08 | 0.70 | 1.44 |
| LDMC | -1.69 | 0.10 | 0.62 | 1.61 |
| LN | 1.40 | 0.17 | 0.77 | 1.29 |
| LP | -1.94 | 0.06 | 0.74 | 1.34 |

Key: LA, area of leaf; SLA, specific Leaf Area; LDMC, leaf dry matter content; LN, leaf nitrogen content; LP, Leaf phosphorus content.
